# Karyotype characterization and comparison of three hexaploid species of *Bromus* Linnaeus, 1753 (Poaceae)

**DOI:** 10.3897/CompCytogen.v11i2.11572

**Published:** 2017-04-07

**Authors:** Leonardo Luís Artico, Ana Cristina Mazzocato, Juliano Lino Ferreira, Carlos Roberto Carvalho, Wellington Ronildo Clarindo

**Affiliations:** 1 Setor de Plantas Forrageiras, Embrapa Pecuária Sul. CEP: 96.401-970, Bagé – RS, Brazil; 2 Laboratório de Citogenética e Citometria, Departamento de Biologia Geral, Centro de Ciências Biológicas e da Saúde, Universidade Federal de Viçosa. CEP: 36.570-000, Viçosa – MG, Brazil; 3 Laboratório de Citogenética e Cultura de Tecidos Vegetais, Departamento de Biologia, Universidade Federal do Espírito Santo. CEP: 29.500-000, Alegre – ES, Brazil

**Keywords:** Karyogram, nuclear genome size, polyploidy, forage grasses

## Abstract

Chromosome morphometry and nuclear DNA content are useful data for cytotaxonomy and to understand the evolutionary history of different taxa. For the genus *Bromus* Linnaeus, 1753, distinct ploidy levels have been reported, occurring from diploid to duodecaploid species. The geographic distribution of *Bromus* species has been correlated with chromosome number and ploidy level. In this study, the aims were to determine the nuclear genome size and characterize the karyotype of the South American *Bromus* species: *Bromus auleticus* Trinius ex Nees, 1829, *Bromus brachyanthera* Döll, 1878 and *Bromus catharticus* Vahl, 1791. The mean nuclear 2C value ranged from 2C = 12.64 pg for *B. catharticus* to 2C = 17.92 pg for *B. auleticus*, meaning a maximum variation of 2C = 5.28 pg, equivalent to 41.70%. Despite this significant difference in 2C value, the three species exhibit the same chromosome number, 2n = 6x = 42, which confirms their hexaploid origin. Corroborating the genome size, the chromosome morphometry (total, short- and long-arm length) and, consequently, the class differed among the karyotypes of the species. Based on the first karyograms for these *Bromus* species, some morphologically similar and several distinct chromosome pairs were found. Therefore, the karyotype characterization confirmed the hexaploid origin of the studied *Bromus* species, which differ in relation to the karyogram and the nuclear 2C value. Considering this, cytogenetics and flow cytometry can be used to discriminate *Bromus* species, contributing to taxonomy and systematic studies and providing information on the evolutionary history of this taxa.

## Introduction

The genus *Bromus* Linnaeus, 1753, family Poaceae comprises more than 160 species of annual and perennial grasses (Acedo and Liamas 2001). This taxon is widely distributed around the world (Williams et al. 2011), demonstrating the adaptability of its species (Martinello and Schifino-Wittmann 2003). The genus *Bromus* includes important forage grasses, such as *Bromus auleticus* Trinius ex Nees, 1829, *Bromus brachyanthera* Döll, 1878 and *Bromus catharticus* Vahl, 1791 (Puecher et al. 2001, Martinello and Schifino-Wittmann 2003, Iannone et al. 2010).

The basic chromosome number of the genus *Bromus* is x = 7, and its species possess karyotypes varying from 2n = 2x = 14 (diploid) to 2n = 12x = 84 (duodecaploid) (Fedorov 1969, Armstrong 1984, 1987, Klos et al. 2009, Williams et al. 2011). Most of the species are diploid (2n = 2x = 14) or tetraploid (2n = 4x = 28) (Martinello and Schifino-Wittmann 2003), but large variation in chromosome number among *Bromus* species has been found, such as: *Bromus cappadocicus* Boissier et Balansa, 1857, and *B. tomentosus* Trinius, 1813, with 2n = 2x = 14; *B. erectus* Huds., 1762, *B. biebersteinii* Roemer et Schultes, 1817, and *B. stenostachyus* Boissier, 1884, with 2n = 4x = 28; *B. tomentellus* Boissier, 1846, *B. variegatus* M. Bieberstein, 1819 (Sheidai et al. 2008), *B. auleticus* (Martinello and Schifino-Wittmann 2003), *B. bonariensis* Parodi et J. H. Camara, 1963, *B. brevis* Steudel, 1854, *B. parodii* Covas et Itria, 1968, *B. brachyanthera* and *B. catharticus* Vahl, 1791 (Schifino and Winge 1983, Naranjo 1985) with 2n = 6x = 42. Variation in chromosome number has also been found within the same species, such as in *Bromus kopetdagensis* Drobow, 1925, (2n = 6x = 42 in Tehran and 2n = 10x = 70 in Emamzadeh-Hashem; Sheidai et al. 2008) and in *Bromus setifolius* J. Presl, 1830, (2n = 10x = 70 for ‘Pictus’ and ‘Brevifolius’, and 2n = 4x = 28 for ‘Setifolius’; García et al. 2009). So, cytogenetic and plant morphology data supported the classification of the *B. setifolius* lines as separate species (García et al. 2009).

Karyotype characterization showed that the chromosomes of the *Bromus* species are similar in relation to total length and class (Joachimiak et al. 2001), with the occurrence of metacentric and submetacentric chromosomes being reported (Sheidai et al. 2008). This way, the distinction between karyotypes of *Bromus* species with the same chromosome number is generally carried out based on the size of the satellite portions (Armstrong 1983, Joachimiak et al. 2001) and heterochromatin distribution (Klos et al. 2009).

Stebbins (1981) reported that the genus *Bromus* originated in Eurasia. During the Pliocene, three sections were originated: *Ceratochloa* P. Beauvois, 1812, *Pnigma* Dumort, 1823 and *Neobromus* Shear, 1900, being that *Ceratochloa* and *Neobromus* spread to Americas. Given this hypothesis, Stebbins argued that the geographic region Eurasia was also the differentiation center of diploid, tetraploid and, most likely, hexaploid species.

Differentiation of allohexaploid *Bromus* species in South America proceeded in the Pleistocene. Meanwhile, in North America, allopolyploidy events also occurred, leading to new ones with higher ploidy level (8x, 12x) (Stebbins 1947, 1981). This way, the *Bromus* species in South America is somewhat restricted to hexaploid species, differently from those found in North America, which are octoploids and duodecaploids (Massa et al. 2004).

According to current knowledge, the South American species (as *B. auleticus*, *B. brachyanthera* and *B. catharticus*) have chromosome number of 2n = 6x = 42 (Schifino and Winge 1983, Naranjo 1985, Martinello and Schifino-Wittmann 2003). Nevertheless, it is still necessary to confirm and understand the chromosomal changes among these species. Because chromosomal changes constitute an important mechanism of diversification and speciation (Stace 2000, Peer et al. 2009, Weiss-Schneeweiss et al. 2013), the investigation of this aspect in *Bromus* species of South America may generate knowledge on the speciation processes in this genus.

Numerical and structural chromosomal rearrangements have been reported to trigger changes in karyotype in various plant taxa. Due to these changes, the nuclear genome size varies between phylogenetically related species (Raskina et al. 2008, Bonifácio et al. 2012). Thus, nuclear DNA content measurements have increasingly been employed in taxonomic, systematic and evolutive approaches using flow cytometry (FCM). In addition to its practicality and reproducibility, FCM is useful to reveal differences among taxa, especially those that exhibit conserved chromosome number (Mabuchi et al. 2005). The nuclear genome size was measured for *Bromus* hexaploid (2n = 6x = 42) species. In spite of the same chromosome number, the seven species showed distinct nuclear 2C values, varying from 2C = 12.72 pg in *Bromus willdenowii* Kunth, 1829, to 2C = 15.10 pg in *Bromus lithobius* Trin., 1836 (Klos et al. 2009).

Hence, karyotype and nuclear 2C value are relevant data for the taxonomy and systematics of *Bromus*, as well as for understanding the evolutionary history of the genus and the relationships within the taxa. Thus, the aims of the present study were to measure the nuclear 2C value, determine the chromosome number and characterize the karyotype of the South American *Bromus* species *B. auleticus*, *B. brachyanthera* and *B. catharticus*.

## Material and methods

### Plant samples

Seeds of *B. auleticus*, *B. brachyanthera* and *B. catharticus* were provided by the South Forage Germplasm Bank (BAG) of Embrapa South Livestock, Brazil (BRA 00059183-4, 00080317-1 and 00059197-4, respectively). The seed samples were collected from several individuals of each species, occurring in the Brazilian Pampa biome, state of Rio Grande do Sul, Brazil. Copies of the species were deposited in the Herbarium CNPO Embrapa (voucher numbers CNPO 4408 for *B. auleticus*, CNPO 4412 for *B. brachyanthera*, and CNPO 4408 for *B. catharticus*).

### Nuclear 2C DNA measurement

Nuclear suspensions were prepared from leaf fragments (2 cm^2^) obtained from each specimen of *B. auleticus*, *B. brachyanthera* or *B. catharticus* (samples), together with the internal standard *Pisum sativum* L. (2C = 9.16 pg; Praça-Fontes et al. 2011). For nuclear extraction, chopping was performed in 0.5 mL of OTTO-I buffer (Otto 1990) supplemented with 2 mM dithiothreitol and 50 µg mL^-1^ RNAse. Next, 0.5 mL of OTTO-I buffer was added, and the suspensions were filtered through nylon mesh of 30 µM, placed in microtube and centrifuged at 100 *x*g for 5 min. The pellet was resuspended in 100 μl OTTO-I buffer and incubated for 10 min (Praça-Fontes et al. 2011). The nuclei suspensions were stained with 1.5 ml of OTTO I:OTTO II (1:2 – Otto 1990; Praça-Fontes et al. 2011) supplemented with 2 mM dithiothreitol, 50 μg ml^-1^ propidium iodide and 50 μg ml^-1^ RNase (Praça-Fontes et al. 2011). The suspensions were kept in the dark for 30 min, filtered through a 20-µM nylon mesh, and analyzed on a flow cytometer Partec PAS II / III (Partec GmbH, Germany) equipped with a laser source (488 nm). For determination of the nuclear DNA content, histograms were analyzed with the Max Partec Flow software tools. Six independent repetitions, accounting for more than 10,000 nuclei, were carried out in each analysis. The genome size of *Bromus* species was calculated according to the formula:

**Figure:**
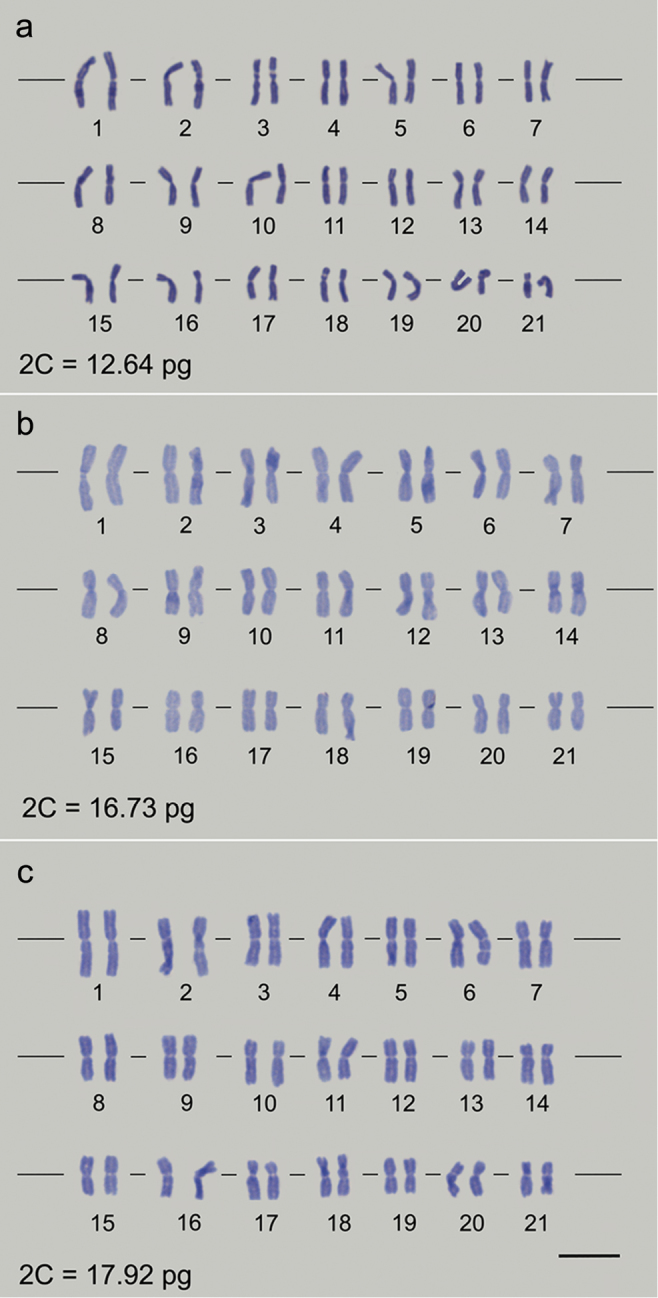


Wherein: 2C_D_: value of 2C DNA content (pg) of each *Bromus* species; C1: average G_0_/G_1_ peak channel of the *Bromus* species; C2: average G_0_/G_1_ peak channel of *P. sativum*; 2C_S_: value of 2C DNA content of *P. sativum* (2C = 9.16 pg).

### Karyotype characterization and morphometric analysis

The seeds were aseptically scarified, disinfested, inoculated into medium composed of half-strength MS salts, 10 ml l^-1^ MS vitamins (Murashige and Skoog 1962), 30 g l^-1^ sucrose and 2.8 g l^-1^ Phytagel, and grown in photoperiod of 16 h at 25 ± 2°C. Roots of the seedlings were excised and treated with 4 µM amiprophos-methyl (APM, Sigma) for 4 h (B.O.D., 30°C). Root apical meristems were washed in dH_2_O, fixed in methanol:acetic acid (Merck) solution (3:1), and stored at -20°C. After 24 h, the meristems were washed in dH_2_O and macerated in enzyme solution pool (96.6% pectinase, 0.4% hemicellulose, 1.0% macerozyme and 4.0% cellulose, Sigma) 1:20 (pool:dH_2_O) for 2 h at 34°C. Again, the meristems were washed with dH_2_O, fixed, and stored at -20°C. Subsequently, slides were prepared by dissociation of the macerated meristems and air-drying (Carvalho et al. 2007). The slides were stained with 5% Giemsa (Merck) for 6 min, washed twice in dH_2_O, and dried under heating plate. All slides were examined under a microscope Nikon Eclipse Ci-S (Nikon). Mitotic images were captured with the objective 100x and CCD camera (Nikon Evolution^TM^) coupled to the microscope. Morphometric analysis of the chromosomes of the three *Bromus* species was performed for the total length, length of the long and short arms, arm ratio, and chromosome class. The latter was determined as proposed by Levan et al. (1964) and reviewed by Guerra (1986).

## Results

FCM nuclear suspensions resulted in G_0_/G_1_ fluorescence peaks with a coefficient of variation of less than 5% for *Bromus* species and *P. sativum*. Thereby, FCM procedures provided suspensions with adequate amount of isolated, intact and stoichiometrically stained nuclei. The 2C nuclear DNA content was measured for the *Bromus* species through analysis of the histograms. 2C value of *B. catharticus* was 2C = 12.64 ± 0.00 pg, *B. brachyanthera* was 2C = 16.73 ± 0.16 pg, and *B. auleticus* was 17.92 ± 0.44 pg. Mean value for *B. auleticus* was 41.70% higher than for *B. catharticus*, and 7.10% greater than for *B. brachyanthera*. In turn, *B. brachyanthera* presented 2C value 32.36% higher than that of *B. catharticus*. These values reflect interspecific variation among the nuclear genome sizes of the analyzed species.

**Figure 1.**
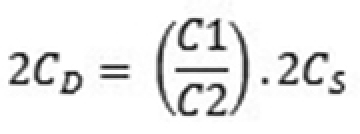
First karyograms of *Bromus* species, displaying 2n = 6x = 42 chromosomes: **a** *B. catharticus* **b** *B. brachyanthera* and **c** *B. auleticus*. **a** *B. catharticus* displayed eleven metacentric (1, 4, 5, 7, 8, 9, 10, 11, 14, 17 and 20) and ten submetacentric chromosomes (2, 3, 6, 12, 13, 15, 16, 18, 19 and 21) **b** *B. brachyanthera* exhibited 13 metacentric (1, 2, 4, 5, 6, 9, 10, 13, 15, 16, 17, 19 and 21) and eight submetacentric chromosomes (3, 7, 8, 11, 12, 14, 18 and 20) **c** *B. auleticus* presented eleven metacentric (1, 3, 4, 5, 6, 8, 9, 11, 15, 18 and 19) and ten submetacentric chromosomes (2, 7, 10, 12, 13, 14, 16, 17, 20 and 21). Note the morphologically similar chromosomes: **a** 12–13 in *B. catharticus* **b** 11–12 and 15–16 in *B. brachyanthera*, and **c** 3–4 in *B. auleticus*. Bar = 5 µm.

Root meristems treated with amiprophos-methyl and macerated in enzyme pool solution resulted in adequate metaphase chromosomes. Metaphases were chosen based on the following criteria: well-spread chromosomes with well-defined constriction, no chromatin deformations and no cytoplasmic background noise. These features allowed accurate chromosome counting, karyotype measurements, chromosome class determination and karyogram assembly. All three *Bromus* species showed a conserved number of 2n = 42 chromosomes (Figure 1).

Based on the morphometric data, the chromosome class was determined and the differences between the karyotypes of the three species were verified. *B. auleticus* presented eleven metacentric (1, 3, 4, 5, 6, 8, 9, 11, 15, 18 and 19) and ten submetacentric chromosomes (2, 7, 10, 12, 13, 14, 16, 17, 20 and 21). *B. brachyanthera* exhibited 13 metacentric (1, 2, 4, 5, 6, 9, 10, 13, 15, 16, 17, 19 and 21) and eight submetacentric chromosomes (3, 7, 8, 11, 12, 14, 18 and 20). Finally, *B. catharticus* displayed eleven metacentric (1, 4, 5, 7, 8, 9, 10, 11, 14, 17 and 20) and ten submetacentric chromosomes (2, 3, 6, 12, 13, 15, 16, 18, 19 and 21) (Table 1). Groups of morphologically similar chromosome pairs were also identified for all *Bromus* species: 3–4 in *B. auleticus*, 11–12 and 15–16 in *B. brachyanthera*, and 12–13 in *B. catharticus* (Figure 1, Table 1).

The three *Bromus* species presented only metacentric and submetacentric chromosomes. Despite belonging to the same class, the chromosomes differed intra- and interspecifically based on their morphology, which was characterized by occurrence of well-defined telomere and centromere portions and relatively low chromatin compaction level (Figure 1). As summarized in Table 1, the majority of the chromosomes could be distinguished by at least one morphometric parameter: total length, short- and long-arm length, ratio between arms, and/or relative chromosome size (%) in relation to the sum of the total length (Table 1). In addition, some chromosomes showed the secondary constriction in the interstitial region of the short arm, such as the chromosome 3 of *B. catharticus* (Figure 1a) or 18 of *B. auleticus* (Figure 1c).

**Table 1.** Morphometry of the metaphasic chromosomes of *B. auleticus*, *B. brachyanthera* and *B. catharticus*.

Chrom.	*B. auleticus*						*B. brachyanthera*						*B. catharticus*					
	Total (µm)	Arms		r	Class	Size (%)	Total (µm)	Arms		r	Class	Size (%)	Total	Arms		r	Class	Size (%)
		Short	Long					Short	Long				(µm)	Short	Long			
1	5.867	2.667	3.200	1.20	M	7.55	5.233	2.467	2.767	1.12	M	6.41	4.567	2.233	2.333	1.04	M	6.96
2	4.833	1.733	3.100	1.79	SM	6.22	4.767	2.133	2.633	1.23	M	5.84	3.833	1.500	2.333	1.56	SM	5.85
3	4.333	2.067	2.267	1.10	M	5.58	4.733	1.867	2.867	1.54	SM	5.80	3.800	1.500	2.300	1.53	SM	5.80
4	4.333	1.967	2.367	1.20	M	5.58	4.533	1.900	2.633	1.39	M	5.55	3.700	1.633	2.067	1.27	M	5.64
5	4.100	1.867	2.233	1.20	M	5.28	4.500	2.067	2.433	1.18	M	5.51	3.467	1.567	1.900	1.21	M	5.29
6	3.967	1.700	2.267	1.33	M	5.11	4.100	1.967	2.133	1.08	M	5.02	3.467	1.367	2.100	1.54	SM	5.29
7	3.900	1.433	2.467	1.72	SM	5.02	4.000	1.433	2.567	1.79	SM	4.90	3.400	1.667	1.733	1.04	M	5.19
8	3.900	1.667	2.233	1.34	M	5.02	3.933	1.500	2.433	1.62	SM	4.82	3.367	1.600	1.767	1.10	M	5.13
9	3.733	1.667	1.967	1.18	M	4.68	3.900	1.933	1.967	1.02	M	4.78	3.233	1.300	1.933	1.49	M	4.93
10	3.633	1.400	2.333	1.67	SM	4.80	3.867	1.833	2.033	1.11	M	4.74	3.167	1.567	1.600	1.02	M	4.83
11	3.600	1.633	1.967	1.20	M	4.63	3.800	1.467	2.333	1.59	SM	4.66	3.100	1.300	1.800	1.38	M	4.73
12	3.567	1.333	2.233	1.68	SM	4.59	3.800	1.467	2.333	1.59	SM	4.66	3.067	1.210	1.857	1.53	SM	4.68
13	3.333	1.300	2.033	1.56	SM	4.29	3.600	1.567	2.033	1.30	M	4.41	2.967	1.170	1.797	1.54	SM	4.52
14	3.333	1.100	2.233	2.03	SM	4.29	3.467	1.367	2.100	1.54	SM	4.25	2.967	1.267	1.700	1.34	M	4.52
15	3.333	1.567	1.767	1.13	M	4.29	3.433	1.533	1.900	1.24	M	4.21	2.800	1.033	1.767	1.71	SM	4.27
16	3.300	1.300	2.000	1.54	SM	4.25	3.433	1.500	1.933	1.29	M	4.21	2.733	0.867	1.867	2.15	SM	4.17
17	3.167	1.100	2.067	1.88	SM	4.08	3.400	1.533	1.867	1.22	M	4.17	2.733	1.300	1.433	1.10	M	4.17
18	3.000	1.467	1.533	1.05	M	3.86	3.400	1.200	2.200	1.83	SM	4.17	2.700	1.067	1.633	1.53	SM	4.12
19	2.933	1.300	1.633	1.26	M	3.78	3.300	1.500	1.800	1.20	M	4.04	2.367	0.800	1.567	1.96	SM	3.61
20	2.833	1.133	1.700	1.50	SM	3.65	3.267	1.033	2.233	2.16	SM	4.00	2.267	0.933	1.333	1.43	M	3.46
21	2.700	1.067	1.633	1.53	SM	3.47	3.167	1.300	1.867	1.44	M	3.88	1.867	0.600	1.267	2.11	SM	2.85
Sum	77.700	32.470	45.230	-	-	100.00	81.630	34.570	47.070	-	-	100.00	65.570	27.480	38.090	-	-	100.00

Chrom – chromosome; r – arm ratio (long/short); Size – % size in relation to sum of the mean values of total length; M – metacentric; SM – submetacentric; Sum – sum of the mean values.

## Discussion

According to the chromosome number found here and the complement set x = 7 (Stebbins 1981), *B. auleticus*, *B. brachyanthera* and *B. catharticus* are hexaploid species (2n = 6x = 42). This result is in accordance with previous data reported for the three species (Schifino and Winge 1983, Naranjo 1985, Martinello and Schifino-Wittmann 2003). The genus *Bromus* originated in Eurasia, whereas the hexaploid species emerged in South America during the Pleistocene, from the subgenus Ceratochloa (Stebbins 1981). Thus, the chromosome number of the three species has remained conserved in relation to the ancestors, supporting Stebbins’s hypothesis on the diversification of the *Bromus* species.

The mean nuclear 2C value divergences were corroborated by chromosome morphometry. *B. catharticus* clearly differed from the other species, being that its relatively small genome size correlated with the sum of the total chromosome length. Differently, for *B. auleticus* and *B. brachyanthera* this relation was not observed, as a result of the low compaction level of the chromatin in the latter species (Figure 1, Table 1). The large differences among nuclear 2C values for the same chromosome number suggests that the three species diverged through chromosomal rearrangements. In a study of hexaploid *Bromus* species, Klos et al. (2009) also reported interspecific variations (2C = 12.72 to 15.10 pg) in relation to the nuclear genome size. According to these authors, chromosomal changes occurred during the evolution of the hexaploid *Bromus* species, most likely through the gain or loss of highly repeated DNA sequences. Regarding this, karyotype and nuclear genome size should be considered together when comparing *Bromus* species.

Despite the similar morphology of some chromosome pairs (12–13 in *B. catharticus*, 11–12 and 15–16 in *B. brachyanthera*, and 3–4 in *B. auleticus*; Figure 1, Table 1), differences were found for most pairs of the three hexaploid *Bromus* species (Figure 1, Table 1). For the genus *Bromus*, Stebbins (1981) classified all species as allohexaploids. The allopolyploidy in *Bromus* was also found in *Bromus hordeaceus* L., 1753, which was classified as an allotetraploid (Ainouche et al. 1999). In that sense, Klos et al. (2009) highlighted that the Bromus section Ceratochloa includes a number of closely related allopolyploid species originated by three ancestors AABBCC.

## Conclusion

The nuclear 2C value and karyotype characterization allowed differentiating the three *Bromus* species, thus contributing to the cytotaxonomy and evolutional understanding in this taxon. As also demonstrated by other authors, these data provide insights about the evolutionary process and diversification of the polyploid *Bromus* species.

## References

[B1] Acedo C Liamas F (2001) Variation of morphological characters of lemma and palea in the genus *Bromus* (Poaceae). Annales Botanici Fennici 38: 1–14. https://doi.org/10.2307/i23726794

[B2] Ainouche ML Bayer RJ Gourret JP Defontaine A Misset MT (1999) The allotetraploid invasive weed *Bromus hordeaceus* L. (Poaceae): genetic diversity, origin and molecular evolution. Folia Geobot 34: 405–419. https://doi.org/10.1007/BF02914919

[B3] Armstrong KC (1983) The relationship between some Eurasian and American species of Bromus section Pnigma as determined by the karyotypes of some F1 hybrids. Canadian Journal of Plant Science 61: 700–707. https://doi.org/10.1139/b83-080

[B4] Armstrong KC (1984) Chromosome pairing affinities between Old and New World species of Bromus section Pnigma. Canadian Journal of Plant Science 62: 581–585.

[B5] Armstrong KC (1987) Chromosome numbers of perennial *Bromus* species collected in the USSR. Canadian Journal of Plant Science 67: 267–269. https://doi.org/10.4141/cjps87-038

[B6] Bonifácio EM Fonsêca A Almeida C Santos KGB Pedrosa-Harand A (2012) Comparative cytogenetic mapping between the lima bean (*Phaseolus lunatus* L.) and the common bean (*P. vulgaris* L.). Theoretical and Applied Genetics 124: 1513–1520. https://doi.org/10.1007/s00122-012-1806-x 22331139 10.1007/s00122-012-1806-x

[B7] Carvalho CR Clarindo WR Almeida PM (2007) Plant cytogenetics: still looking for the perfect mitotic chromosomes. Nucleus 50: 453–462.

[B8] Fedorov AA (1969) Chromosome Numbers of Flowering Plants. Russian Academy of Sciences, Leningrad, 926 pp.

[B9] García AM Schrauf GE González G Poggio L Naranjo CA Dupal MP Spangenberg GC Forster JW (2009) Use of AFLP and RAPD molecular genetic markers and cytogenetic analysis to explore relationships among taxa of the Patagonian *Bromus setifolius* complex. Genetics and Molecular Biology 32: 312–319. https://doi.org/10.1590/S1415-47572009005000029 21637686 10.1590/S1415-47572009005000029 PMC3036919

[B10] Guerra MS (1986) Reviewing the chromosome nomenclature of Levan et al. Revista Brasileira de Genética 9: 741–743.

[B11] Iannone LJ WhiteJr JF Giussani LM Cabral D Novas MV (2010) Diversity and distribution of *Neotyphodium*-infected grasses in Argentina. Mycological Progress 10: 9. https://doi.org/10.1007/s11557-010-0669-2

[B12] Joachimiak A Kula A Sliwinska E Sobieszczanska A (2001) C-banding and nuclear DNA amount in six *Bromus* species. Acta Biologica Cracoviensia Series Botanica 43: 105–115.

[B13] Klos J Sliwinska E Kula A Golczyk H Grabowska-Joachimiak A Ilnicki T Szostek K Stewart A Joachimiak AJ (2009) Karyotype and nuclear DNA content of hexa-, octo-, and duodecaploid lines of Bromus subgen. Ceratochloa. Genetics and Molecular Biology, 32: 528–537. https://doi.org/10.1590/S141547572009005000046 21637516 10.1590/S1415-47572009005000046 PMC3036049

[B14] Levan A Fredga K Sandberg AA (1964) Nomenclature for centromeric position on chromosomes. Hereditas 52: 201–220. https://doi.org/10.1111/j.1601-5223.1964.tb01953.x

[B15] Mabuchi T Kokubun H Mii M Ando T (2005) Nuclear DNA content in the genus *Hepatica* (Ranunculaceae). Journal of Plant Research 118: 37–41. https://doi.org/10.1007/s10265-005-0191-9 15711889 10.1007/s10265-005-0191-9

[B16] Martinello GE Schifino-Wittmann MT (2003) Chromosomes of *Bromus auleticus* Trin. ex Nees (Poaceae). Genetics and Molecular Biology 26: 369–371. https://doi.org/10.1590/S1415-47572003000300024

[B17] Massa AN Jensen KB Larson SR Hole DJ (2004) Morphological variation in Bromus sect. Ceratochloa germplasm of Patagonia. Canadian Journal of Plant Science 82: 136–144.

[B18] Murashige T Skoog FA (1962) A revised médium for a rapid growth and bioassays with tobacco tissues cultures. Plant Physiol 15: 473–479. https://doi.org/10.1111/j.1399-3054.1962.tb08052.x

[B19] Naranjo CA (1985) Estudios citogeneticos, bioquimicos y sistemáticos en algunas especies americanas del genero *Bromus* (Gramineae). PhD Thesis, Universidade de Buenos Aires, Buenos Aires.

[B20] Otto FJ (1990) DAPI staining of fixed cells for high-resolution flow cytometry of nuclear DNA. In: Darzynkiewiez Z Crissman HA Robinson JP (Eds) Methods in cell biology. San Diego Academic Press, San Diego, Canadá, 105–110. https://doi.org/10.1016/s0091-679x(08)60516-6 10.1016/s0091-679x(08)60516-6 1707478

[B21] Peer YV Maere S Meyer A (2009) The evolutionary significance of ancient genome duplications. Nature Reviews Genetics 10: 725–732. https://doi.org/10.1038/nrg2600 10.1038/nrg2600 19652647

[B22] Puecher L Claudio G Robredo L Ra´ul D Rios L Rimieri P (2001) Genetic variability measures among *Bromus catharticus* Vahl. Populations and cultivars with RAPD and AFLP markers. Euphytica 121: 229–236. https://doi.org/10.1023/A:1012068415647

[B23] Praça-Fontes MM Carvalho CR Clarindo WR Cruz CD (2011) Revisiting the DNA C-values of the genome size-standards used in plant flow cytometry to choose the ‘‘best primary standards’’. Plant Cell Reports 30: 1183–1191. https://doi.org/10.1007/s00299-011-1026-x 21318354 10.1007/s00299-011-1026-x

[B24] Raskina O Barber JC Nevo E Belyayev A (2008) Repetitive DNA and chromosomal rearrangements: speciation-related events in plant genomes. Cytogenetic and Genome Research 120: 351–377. https://doi.org/10.1159/000121084 18504364 10.1159/000121084

[B25] Schifino MT Winge H (1983) Karyotypes and nuclear DNA content of species of the *Briza* complex and some other genera of Poeae (Gramineae). Revista Brasileira de Genética 6: 245–259.

[B26] Sheidai M Saeidi S Atri M (2008) Taxonomic applications of seed proteins in the genus *Bromus* L. (Poaceae). Iran Journal of Botanical 14: 126–131.

[B27] Stace CA (2000) Cytology and cytogenetics as a fundamental taxonomic resource for the 20th and 21” centuries. Taxon 49: 451–477. https://doi.org/10.2307/1224344

[B28] Stebbins GL (1947) The origin of the complex of *Bromus carinatus* and its phylogeographic implications. Contributions from the Gray Herbarium of Harvard University 165: 42–55.

[B29] Stebbins GL (1981) Chromosomes and evolution in the genus *Bromus* (Gramineae). Botanische Jahrbücher für Systematik, Pflanzengeschichte und Pflanzengeographie 102: 359–379.

[B30] Weiss-Schneeweiss H Emadzade K Jang TS Schneeweiss GM (2013) Evolutionary consequences, constraints and potential of polyploidy in plants. Cytogenetic and Genome Research 140: 137–150. https://doi.org/10.1159/000351727 23796571 10.1159/000351727 PMC3859924

[B31] Williams WM Stewart AV Williamson ML (2011) *Bromus* Wild crop relatives: genomic and breeding resources, millets and grasses. In: Kole C (Ed.) Springer-Verlag Berlin Heidelberg, 15–30. https://doi.org/10.1007/978-3-642-14255-0_2

